# Romero DE, Freitez A, Maia LR, Souza NA. Self-rated health and sociodemographic inequalities among Venezuelan adults: a study based on the *National Survey of Living Conditions* (ENCOVI 2021). Cad Saúde Pública 2024; 40(6):e00149323.

**DOI:** 10.1590/0102-311XEREN149323

**Published:** 2025-02-07

Where it reads:

**Table 2:** Prevalence of Venezuelan adults with fair/poor self-rated health, according to demographic and socioeconomic characteristics. Venezuela, 2021.

Variables	%	95%CI	p-value
Total	17.8	17.2-18.4	
Sex			< 0.001
Male	14.9	14.0-15.8	
Female	19.4	18.6-20.1	
Age (years)			< 0.001
18-29	7.7	6.7-8.7	
30-59	15.6	14.9-16.3	
60 or more	32.9	31.3-34.5	
Education			< 0.001
Complete primary education or less	24.6	23.2-25.9	
Incomplete high school	19.1	17.5-20.7	
Complete high school	15.0	13.9-16.0	
Incomplete higher education or more	12.3	11.3-13.3	
Level of monetary poverty			< 0.001
Not poor	12.6	10.8-14.4	
Not extreme poverty	17.0	15.8-18.2	
Extreme poverty	18.7	17.9-19.4	
Food Insecurity Scale			< 0.001
No food insecurity	9.4	7.7-11.2	
Mild food insecurity	12.7	11.8-13.6	
Moderate food insecurity	20.0	18.9-21.1	
Severe food insecurity	23.8	22.5-25.1	
Parental emigration			< 0.001
No	17.3	16.7-17.9	
Yes	22,7	20.5-24.9	

I95%CI: 95% confidence interval.

Source: *National Survey of Living Conditions* (ENCOVI 2021) ^9^.

**Table 3:** Crude and adjusted prevalence ratios (PR) of fair/poor self-rated health, according to demographic and socioeconomic characteristics in adults. Venezuela, 2021.

Variables	Crude PR	95%CI	p-value	Adjusted PR	95%CI	p-value
Sex						
Male	1.41	1.31-1.53	< 0.001	1.35	1.25-1.47	< 0.001
Female	1.00	-	-	1.00		
Age (years)						
18-29	4.10	3.57-4.71	< 0.001	3.81	3.29-4.41	< 0.001
30-59	1.97	1.72-2.25	< 0.001	1.91	1.66-2.20	< 0.001
60 or more	1.00	-	-	1.00		
Education						
Complete primary education or less	1.45	1.31-1.62	< 0.001	1.36	1.22-1.53	< 0.001
Incomplete high school	1.57	1.39-1.76	< 0.001	1.40	1.23-1.58	< 0.001
Complete high school	1.30	1.17-1.46	< 0.001	1.19	1.06-1.33	0.003
Incomplete higher education or more	1.00	-	-	1.00		
Level of monetary poverty			-			
Not poor	1.84	1.57-2.15	< 0.001	1.19	1.01-1.41	0.042
Not extreme poverty	1.49	1.26-1.77	< 0.001	1.13	0.95-1.35	0.173
Extreme poverty	1.00	-	-	1.00		
Food Insecurity Scale						
No food insecurity	2.23	1.83-2.71	< 0.001	2.00	1.61-2.47	< 0.001
Mild food insecurity	2.04	1.67-2.47	< 0.001	1.85	1.50-2.28	< 0.001
Moderate food insecurity	1.30	1.07-1.59	0.009	1.27	1.03-1.57	0.027
Severe food insecurity	1.00			1.00		
Parental emigration						
No	1.20	1.08-1.34	0.001	1.32	1.18-1.48	< 0.001
Yes	1.00	-		1.00		

I95%CI: 95% confidence interval.

Source: *National Survey of Living Conditions* (ENCOVI 2021) ^9^.

It shoul read:

**Table 2:** Prevalence of Venezuelan adults with fair/poor self-rated health, according to demographic and socioeconomic characteristics. Venezuela, 2021.

Variables	%	95%CI	p-value
Total	17.8	17.2-18.4	
Sex			< 0.001
Male	14.9	14.0-15.8	
Female	19.4	18.6-20.1	
Age (years)			< 0.001
18-29	7.7	6.7-8.7	
30-59	15.6	14.9-16.3	
60 or more	32.9	31.3-34.5	
Education			< 0.001
Complete primary education or less	24.6	23.2-25.9	
Incomplete high school	19.1	17.5-20.7	
Complete high school	15.0	13.9-16.0	
Incomplete higher education or more	12.3	11.3-13.3	
Level of monetary poverty			< 0.001
Not poor	12.6	10.8-14.4	
Not extreme poverty	17.0	15.8-18.2	
Extreme poverty	18.7	17.9-19.4	
Food Insecurity Scale			< 0.001
No food insecurity	9.4	7.7-11.2	
Mild food insecurity	12.7	11.8-13.6	
Moderate food insecurity	20.0	18.9-21.1	
Severe food insecurity	23.8	22.5-25.1	
Parental emigration			< 0.001
No	17.3	16.7-17.9	
Yes	22.7	20.5-24.9	

95%CI: 95% confidence interval.

Source: *National Survey of Living Conditions* (ENCOVI 2021) ^9^.

**Table 3:** Crude and adjusted prevalence ratios (PR) of fair/poor self-rated health, according to demographic and socioeconomic characteristics in adults. Venezuela, 2021.

Variables	Crude PR	95%CI	p-value	Adjusted PR	95%CI	p-value
Sex						
Male	1.00	-	-	1.00	-	-
Female	1.41	1.31-1.53	< 0.001	1.35	1.25-1.47	< 0,001
Age (years)						
18-29	1.00	-	-	1.00	-	-
30-59	1.97	1.72-2.25	< 0,001	1.91	1.66-2.20	< 0.001
60 or more	4.10	3.57-4.71	< 0.001	3.81	3.29-4.41	< 0.001
Education						
Complete primary education or less	1.45	1.31-1.62	< 0.001	1.36	1.22-1.53	< 0.001
Incomplete high school	1.57	1.39-1.76	< 0.001	1.40	1.23-1.58	< 0.001
Complete high school	1.30	1.17-1.46	< 0.001	1.19	1.06-1.33	0.003
Incomplete higher education or more	1.00	-	-	1.00		
Level of monetary poverty			-			
Not poor	1.00	-	-	1.00	-	-
Not extreme poverty	1.49	1.26-1.77	< 0.001	1.13	0.95-1.35	0.173
Extreme poverty	1.84	1.57-2.15	< 0.001	1.19	1.01-1.41	0.042
Food Insecurity Scale						
No food insecurity	1.00	-	-	1.00	-	-
Mild food insecurity	1.30	1.07-1.59	0.009	1.27	1.03-1.57	0.027
Moderate food insecurity	2.04	1.67-2.47	< 0.001	1.85	1.50-2.28	< 0.001
Severe food insecurity	2.23	1.83-2.71	< 0.001	2.00	1.61-2.47	< 0.001
Parental emigration						
No	1.00	-	-	1.00	-	-
Yes	1.20	1.08-1.34	0.001	1.32	1.18-1.48	< 0.001

95%CI: 95% confidence interval.

Source: *National Survey of Living Conditions* (ENCOVI 2021) ^9^.

