# Supplementary material for: Romero DE, Freitez A, Maia LR, Souza NA. Self-rated health and sociodemographic inequalities among Venezuelan adults: a study based on the National Survey of Living Conditions (ENCOVI 2021). Cad Saúde Pública 2024; 40(6):e00149323
Source: Cad Saude Publica. 2025 Feb 7;40(12):eER149323. doi: 10.1590/0102-311XEREN149323 (PMC11805516; doi:10.1590/0102-311XEREN149323)
Supplement: Related texts

# Correction: Romero DE, Freitez A, Maia LR, Souza NA. Autoavaliação de saúde e desigualdades sociodemográficas entre adultos venezuelanos: um estudo com base na *Pesquisa Nacional de Condições de Vida* (ENCOVI 2021). Cad Saúde Pública 2024; 40(6):e00149323.

**DOI:** 10.1590/0102-311XERPT149323

Onde se lê:

**Tabela 1:** Distribuição percentual da população adulta venezuelana segundo características demográficas e socioeconômicas. Venezuela, 2021.

Variáveis	n	%	IC95%
Sexo			
Masculino	5.471	35,2	34,5-36,0
Femininor	10.066	64,8	64,0-65,5
Faixa etária (anos)			
18-29	2.895	18,6	18,0-19,2
30-59	9.368	60,3	59,5-61,1
60 ou mais	3.275	21,1	20,4-21,7
Escolaridade			
Sem escolaridade	60	0,4	0,3-0,5
Primário incompleto	1.505	10,1	9,6-10,6
Primário completo	2.187	14,7	14,1-15,3
Médio incompleto	2.321	15,6	14,7-16,2
Médio completo	4.642	31,2	30,5-32
Superior técnico (incompleto ou completo)	1.184	8,0	7,5-8,4
Superior universitário (incompleto ou completo)	2.963	19,9	19,3-20,6
Nível de pobreza monetária			
Não pobre	1.317	8,5	8,1-9,5
Pobreza não extrema	3.480	22,6	21,9-23,2
Pobreza extrema	10.631	68,9	68,2-69,6
Escala de Insegurança Alimentar			
Sem insegurança alimentar	1.093	7,0	6,6-7,4
Insegurança alimentar leve	5.264	33,9	33,2-35,1
Insegurança alimentar moderada	5.172	33,3	32,6-34,1
Insegurança alimentar severa	3.992	25,7	25,0-26,4
Migração parental			
Não	14.105	90,8	90,4-91,3
Sim	1.425	9,2	8,7-9,6
Autoavaliação da saúde			
Muito boa	6.011	38,7	38,0-39,4
Boa	6.763	43,5	42,9-44,2
Regular	2.554	16,4	16,0-16,9
Ruim	209	1,3	1,2-1,5

IC95%: intervalo de 95% de confiança.

Fonte: *Pesquisa Nacional de Condições de Vida* (ENCOVI-2021) ^9^.

**Tabela 3:** Razões de prevalência (RP) brutas e ajustadas da autoavaliação da saúde regular/ruim, segundo características demográficas e socioeconômicas em pessoas adultas. Venezuela, 2021.

Variáveis	RP bruta	IC95%	Valor de p	RP ajustada	IC95%	Valor de p
Sexo						
Masculino	1,41	1,31-1,53	< 0,001	1,35	1,25-1,47	< 0,001
Feminino	1,00	-	-	1,00		
Faixa etária (anos)						
18-29	4,10	3,57-4,71	< 0,001	3,81	3,29-4,41	< 0,001
30-59	1,97	1,72-2,25	< 0,001	1,91	1,66-2,20	< 0,001
60 ou mais	1,00	-	-	1,00		
Escolaridade						
Primário completo ou menos	1,45	1,31-1,62	< 0,001	1,36	1,22-1,53	< 0,001
Médio incompleto	1,57	1,39-1,76	< 0,001	1,40	1,23-1,58	< 0,001
Médio completo	1,30	1,17-1,46	< 0,001	1,19	1,06-1,33	0,003
Superior incompleto ou mais	1,00	-	-	1,00		
Nível de pobreza monetária			-			
Não pobre	1,84	1,57-2,15	< 0,001	1,19	1,01-1,41	0,042
Pobreza não extrema	1,49	1,26-1,77	< 0,001	1,13	0,95-1,35	0,173
Pobreza extrema	1,00	-	-	1,00		
Escala de Insegurança Alimentar						
Sem insegurança alimentar	2,23	1,83-2,71	< 0,001	2,00	1,61-2,47	< 0,001
Insegurança alimentar leve	2,04	1,67-2,47	< 0,001	1,85	1,50-2,28	< 0,001
Insegurança alimentar moderada	1,30	1,07-1,59	0,009	1,27	1,03-1,57	0,027
Insegurança alimentar severa	1,00			1,00		
Migração parental						
Não	1,20	1,08-1,34	0,001	1,32	1,18-1,48	< 0,001
Sim	1,00	-		1,00		

IC95%: intervalo de 95% de confiança.

Fonte: *Pesquisa Nacional de Condições de Vida* (ENCOVI-2021) ^9^.

Leia-se:

**Tabela 1:** Distribuição percentual da população adulta venezuelana segundo características demográficas e socioeconômicas. Venezuela, 2021.

Variáveis	n	%	IC95%
Sexo			
Masculino	5.471	35,2	34,5-36,0
Feminino	10.066	64,8	64,0-65,5
Faixa etária (anos)			
18-29	2.895	18,6	18,0-19,2
30-59	9.368	60,3	59,5-61,1
60 ou mais	3.275	21,1	20,4-21,7
Escolaridade			
Sem escolaridade	60	0,4	0,3-0,5
Primário incompleto	1.505	10,1	9,6-10,6
Primário completo	2.187	14,7	14,1-15,3
Médio incompleto	2.321	15,6	14,7-16,2
Médio completo	4.642	31,2	30,5-32,0
Superior técnico (incompleto ou completo)	1.184	8,0	7,5-8,4
Superior universitário (incompleto ou completo)	2.963	19,9	19,3-20,6
Nível de pobreza monetária			
Não pobre	1.317	8,5	8,1-9,5
Pobreza não extrema	3.480	22,6	21,9-23,2
Pobreza extrema	10.631	68,9	68,2-69,6
Escala de Insegurança Alimentar			
Sem insegurança alimentar	1.093	7,0	6,6-7,4
Insegurança alimentar leve	5.264	33,9	33,2-35,1
Insegurança alimentar moderada	5.172	33,3	32,6-34,1
Insegurança alimentar severa	3.992	25,7	25,0-26,4
Migração parental			
Não	14.105	90,8	90,4-91,3
Sim	1.425	9,2	8,7-9,6
Autoavaliação da saúde			
Muito boa	6.011	38,7	38,0-39,4
Boa	6.763	43,5	42,9-44,2
Regular	2.554	16,4	16,0-16,9
Ruim	209	1,3	1,2-1,5

IC95%: intervalo de 95% de confiança.

Fonte: *Pesquisa Nacional de Condições de Vida* (ENCOVI-2021) ^9^.

**Tabela 3:** Razões de prevalência (RP) brutas e ajustadas da autoavaliação da saúde regular/ruim, segundo características demográficas e socioeconômicas em pessoas adultas. Venezuela, 2021.

Variáveis	RP bruta	IC95%	Valor de p	RP ajustada	IC95%	Valor de p
Sexo						
Masculino	1,00	-	-	1,00	-	-
Feminino	1,41	1,31-1,53	< 0,001	1,35	1,25-1,47	< 0,001
Faixa etária (anos)						
18-29	1,00	-	-	1,00	-	-
30-59	1,97	1,72-2,25	< 0,001	1,91	1,66-2,20	< 0,001
60 ou mais	4,10	3,57-4,71	< 0,001	3,81	3,29-4,41	< 0,001
Escolaridade						
Primário completo ou menos	1,45	1,31-1,62	< 0,001	1,36	1,22-1,53	< 0,001
Médio incompleto	1,57	1,39-1,76	< 0,001	1,40	1,23-1,58	< 0,001
Médio completo	1,30	1,17-1,46	< 0,001	1,19	1,06-1,33	0,003
Superior incompleto ou mais	1,00	-	-	1,00	-	-
Nível de pobreza monetária			-			
Não pobre	1,00	-	-	1,00	-	-
Pobreza não extrema	1,49	1,26-1,77	< 0,001	1,13	0,95-1,35	0,173
Pobreza extrema	1,84	1,57-2,15	< 0,001	1,19	1,01-1,41	0,042
Escala de Insegurança Alimentar						
Sem insegurança alimentar	1,00	-	-	1,00	-	-
Insegurança alimentar leve	1,30	1,07-1,59	0,009	1,27	1,03-1,57	0,027
Insegurança alimentar moderada	2,04	1,67-2,47	< 0,001	1,85	1,50-2,28	< 0,001
Insegurança alimentar severa	2,23	1,83-2,71	< 0,001	2,00	1,61-2,47	< 0,001
Migração parental						
Não	1,00	-	-	1,00	-	-
Sim	1,20	1,08-1,34	0,001	1,32	1,18-1,48	< 0,001

IC95%: intervalo de 95% de confiança.

Fonte: *Pesquisa Nacional de Condições de Vida* (ENCOVI-2021) ^9^.
